# Justo Gonzalo (1910–1986): a pioneer of brain dynamics

**DOI:** 10.3389/fnana.2026.1754934

**Published:** 2026-03-18

**Authors:** Isabel Gonzalo-Fonrodona

**Affiliations:** Department of Optics, Faculty of Physics, Universidad Complutense de Madrid, Madrid, Spain

**Keywords:** brain dynamics, central syndrome, cortical gradients, facilitation, inverted perception, Justo Gonzalo, multisensory, perception

## Abstract

The present work offers an overview of the pioneering contributions of the neuroscientist Justo Gonzalo to the study of the human cerebral cortex, within the historical context of his time and in relation to current research. Gonzalo initially trained under Gonzalo Rodríguez Lafora (a disciple of Santiago Ramón y Cajal) and always maintained a close relationship with him and his circle, connecting him to the Spanish Neurological School (Cajal’s school). He also trained in neurology in Austria and Germany (1933–1935). The research he called *brain dynamics* began during the Spanish Civil War (1936–1939) in a military hospital in Valencia, based on the study of patients with war-related brain injuries. Based on physiological criteria, Gonzalo described what he termed *central syndrome* of the cortex: a multisensory and bilaterally symmetrical disorder caused by a unilateral parieto-occipital cortical lesion in an associative area. He brought to light singular perceptual phenomena, and was the first to study inverted or tilted perception (visual, tactile, and auditory), the improvement of perception through increased stimulus intensity or the presence of additional stimuli, as well as other phenomena that remain little known today. These findings and the study of different cortical syndromes led Gonzalo to propose that the specificity of brain functions is gradually distributed throughout the cerebral cortex, giving rise to cortical gradients, whose overlap would result in fairly nonspecific or multisensory adaptive regions. This unitary approach went beyond the rigid cortical parcellation for the anatomical localization of brain functions, and is closely aligned with current studies. The interpretation of the *central syndrome* as analogous to the normal case, but with reduced excitability, led Gonzalo to apply scaling concepts that enabled him to develop formalizations and generalizations. He conducted this research in Spain, under very difficult conditions, with the support of the Cajal Institute. Despite the excellent international reception of his works in Spanish during the 1940s and 1950s, his contributions are scarcely known today due to the lack of timely publications in other languages. The publication of his works in English in 2023 has partially filled this gap.

## 1 Introduction

“*To the memory of the great researcher of the nervous system, Santiago Ramón y Cajal*” (translated from Spanish) are the words used by Justo Gonzalo y Rodríguez-Leal to dedicate his book, whose abbreviated title is *Dinámica Cerebral* (Brain Dynamics). The first and second volumes were published in Spanish in 1945 and 1950, respectively, by the Cajal Institute (Gonzalo, 1945, 1950). This marked the first use of the term “brain dynamics” to describe the macroscopic functioning of the brain’s sensory structures.

Even before completing his medical studies in 1933, Gonzalo trained with Gonzalo Rodríguez Lafora and always had a close relationship with him and his circle. Lafora was a disciple of Santiago Ramón y Cajal, Luis Simarro, and Nicolás Achúcarro, which placed Gonzalo in close relation to the so-called Spanish Neurological School (Cajal’s school). Between 1933 and 1935, he received advanced training in neurology in Austria and Germany. Upon his return to Spain, he began a pioneering study of the human cerebral cortex based on patients with brain injuries sustained during the Spanish Civil War (1936–39). The findings and some of the new concepts he introduced were presented in the aforementioned two-volume work, where he detailed the phenomenology of what he called the *central syndrome* of the cortex, a multisensory and bilateral syndrome caused by a unilateral parieto-occipital cortical lesion in an association area, equidistant from the visual, tactile and auditory projection areas. Since this syndrome could not be explained by the classical theory of brain localization, Gonzalo applied a physiological criterion to the analysis of brain functions. Through a series of findings in sensory and multisensory processing, he revealed a dynamic and unified nature of the cerebral cortical functioning (Gonzalo, 1945, 1950, 2010, 2023). This approach enabled him to go beyond the prevailing view of rigid, modular anatomical localization of brain functions, and to propose a system of macroscale cortical gradients, in which functional specificity is gradually distributed across multiple cortical regions. The overlap of the descents of these gradients would give rise to nonspecific areas capable of neural summation and reorganization, i.e., plasticity (Gonzalo, 1952, 2010, 2023). This framework constituted a new model of brain function localization that accounted for the diversity of cortical syndromes, including *central syndrome*. He interpreted the *central syndrome* as a scale reduction in the excitability of the brain system relative to the normal brain. This led him to apply the concepts of similarity and allometry from dynamic systems theory to this syndrome, which was published posthumously (Gonzalo, 2010, 2023, Suppl. II).

This research was very well received internationally at the time, despite being in Spanish. However, it is currently practically unknown in scientific literature, as the necessary translations were not carried out. Notably, the concept of cortical gradients in humans was later proposed by Goldberg (1989), apparently without awareness of Gonzalo’s earlier work. At present, it is firmly established, on the basis of neuroimaging evidence, as fundamental to understanding sensory organization (Bernhardt et al., 2022; Huntenburg et al., 2018; Jung et al., 2022; Margulies et al., 2016; Samara et al., 2023; Song et al., 2025; Wang et al., 2025). There is a recent review by Nashed et al. (2025). Gonzalo’s research is also closely related to metamodal models of the brain (Pascual-Leone and Hamilton, 2001). Since shortly before the year 2000, several multisensory processes have been described in the literature, some of which are closely related to those observed and studied by Gonzalo (Bolognini et al., 2013; Frassinetti et al., 2005; Holmes, 2007; Martuzzi et al., 2007; Meredith and Stein, 1986; Rowland and Stein, 2007; Rowland et al., 2023; Shams and Kim, 2010; Van der Stoep et al., 2019) to cite just a few examples. It could be said that he was decades ahead of some discoveries. However, even today, many of the phenomena described by Gonzalo and concepts he introduced remain little known. The first English edition of his two-volume book, which includes Supplementary material and is freely accessible (Gonzalo, 2023), now enables wider dissemination of his work.

This article presents a chronological account of Gonzalo’s research on brain dynamics conducted in Spain, along with relevant aspects of his life and scientific milieu. Although some of his findings and theoretical contributions have been described in various articles, the present work aims to offer an overall view of his body of work, with the added novelty of enriching the narrative with some previously unpublished details such as certain historical facts and excerpts from texts by other authors commenting on his research. The impact of the Civil War and the long postwar period is clearly reflected: on the one hand, Gonzalo had the opportunity to study a large number of war-related brain injuries, which led him to new discoveries and conceptual developments; on the other hand, the country was economically and scientifically devastated, with significant obstacles to scientific research. The current relevance of his work is also addressed.

## 2 Materials and methods

The primary material used for this work has been the personal archive of Justo Gonzalo (Cajal Institute, Spanish National Research Council, Madrid), as well as the neurobiology library of the Cajal Institute, which also includes Gonzalo’s personal collection. The published work of Justo Gonzalo has also been a valuable source, along with scientific literature from his own time and subsequent decades.

Excerpts from unpublished private correspondence between Justo Gonzalo and several contemporaries have been included in this article as an additional means of illustrating the reception of Gonzalo’s work. These letters are part of the aforementioned personal archive. Their use complies with fair academic use, and only brief, relevant excerpts have been cited.

An effort has been made to provide an objective foundation for the narrative through appropriate references to Gonzalo’s archive, as well as through short excerpts from publications and letters by various authors.

## 3 Results

### 3.1 Up to the beginning of his brain dynamics research in 1938

**FIGURE 1:**
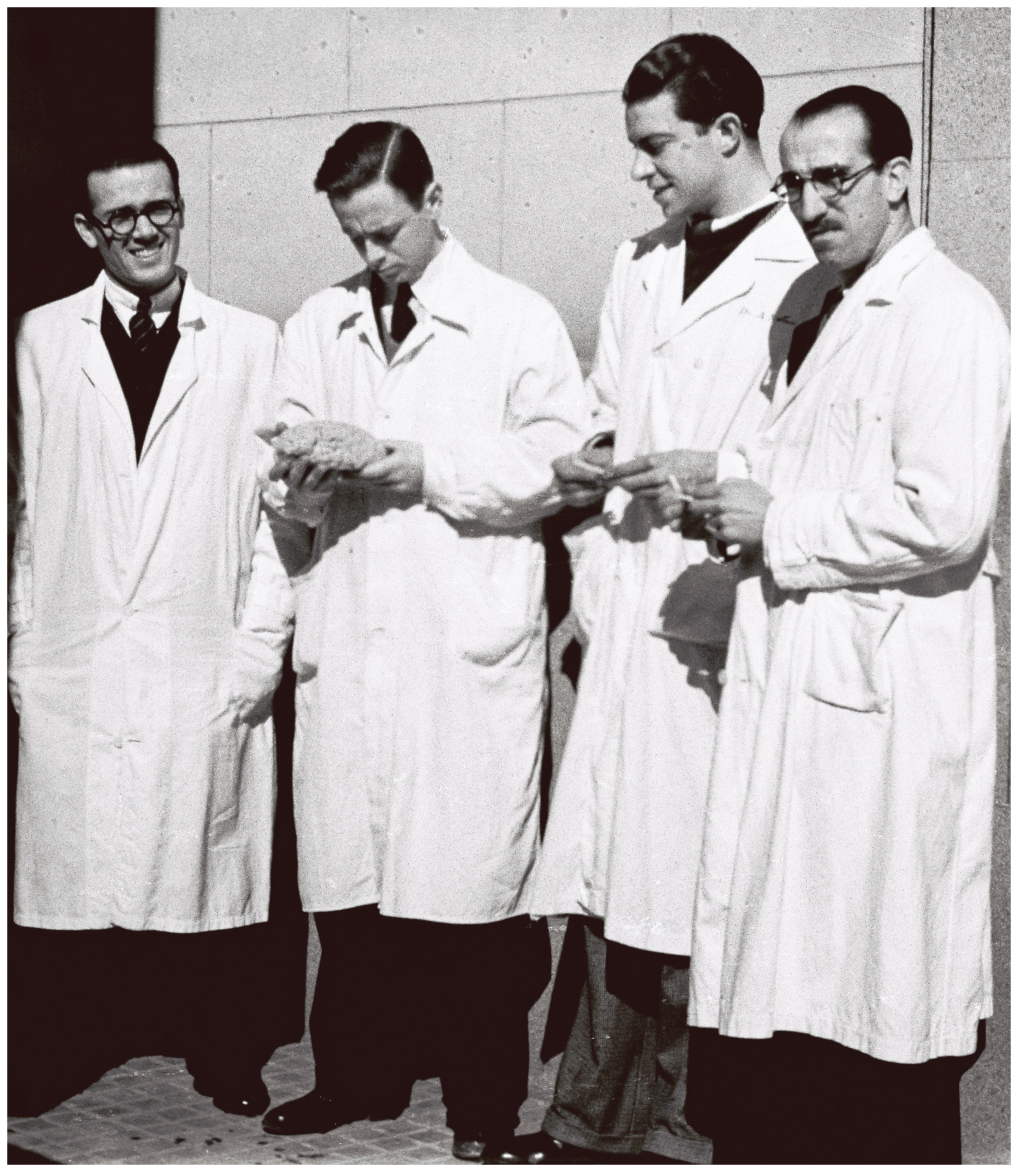
Justo Gonzalo (second from left) at the “Hospital Provincial de Madrid” in 1933. He is looking at a model of the brain that he is holding in his hands. Justo Gonzalo Archive.

Justo Gonzalo was born in 1910 in Barcelona, Spain, as the fourth of ten siblings. From an early age, he showed a keen interest in the nervous system. When his father, a civil engineer, was assigned to Seville, the family moved there and Gonzalo moved to Madrid (closer to Seville) to study medicine. His father died in 1928 at the age of 56, and the family then moved to Madrid, where Gonzalo was studying. Gonzalo’s first histological preparations date back to his student days, made in a small laboratory he had set up at home. During his medical studies, he attended the “Hospital Provincial de Madrid” (Figure 1), in the polyclinic of Gonzalo Rodríguez Lafora, with whom he always maintained a close relationship. After finishing his medical studies in 1933, he continued his neurological training at the University of Vienna (1933–34) in clinical neurology and animal experimentation with Hans Hoff, and in cerebral cytoarchitectonics with Otto Pötzl at the laboratory of Constantin von Economo. Next, with the financial support of the “Junta para Ampliación de Estudios e Investigaciones Científicas” (JAE) (Committee for the Extension of Studies and Scientific Research) he trained in brain pathology with Karl Kleist in Frankfurt (1934–35). As an anecdotal detail, it is worth mentioning what Kleist wrote on the first page of a copy of his book (Kleist, 1934) which he dedicated to Gonzalo in 1935 (a copy from Gonzalo’s library, now housed at the Cajal Institute): “*From this book and from our collaboration, you have drawn knowledge and inspiration with great zeal and understanding, so I am sure that you will be able to accomplish important things in your country, without forgetting German science*” (translated from German).

From 1933 to 1938, Gonzalo published a number of articles (Gonzalo, 1933, 1934a,b, 1935a,b, 1936), and an extensive article together with Kleist on the thalamus (Kleist and Gonzalo, 1938). In 1935, he returned to Madrid and served as a consultant neurologist at the Hospital Provincial de Madrid, where Lafora continued to work, and simultaneously conducted anatomo-clinical studies at the Cajal Institute. In this Hospital, he treated numerous patients wounded in the Spanish Civil War, which erupted in 1936. In 1937, he was assigned as a military physician to the Republican front in the Civil War. The letters he wrote from the military front reflect his concern for his mother and sisters, whose care he entrusted to his great friend, the neurologist Manuel Peraita, who remained in Madrid.

In early 1938, at the request of Lafora, Gonzalo joined the Military Neurological Hospital in Godella (Valencia) as head of the War Neurology Clinic. There, he worked for several months alongside Lafora and the psychiatrist José Miguel Sacristán, with whom he also developed a close friendship, until both Lafora and Sacristán left the hospital. He also worked alongside international volunteers from the United States, members of the International Brigades who had come to support the Republic, such as the neurosurgeon Abraham Ettleson and the physician Barney Malbin.

It was at this hospital, as well as at the hospital in the city of Valencia, where Gonzalo treated a large number of patients with war-related brain injuries, and began a fundamental part of his research. Two patients, among others, were specially studied, those referred to as M, and T, 25 and 20 years old in 1938, respectively, with a left parieto-occipital cortical lesion in an association area, larger in M than in T. In M, the lesion was in the middle of area 19, with probable involvement of the anterior part of area 18 and the most posterior part of area 39, according to Broadman’s terminology. In T, the lesion was practically in the same place but less severe. This type of lesion was referred to as a *central* lesion because of its approximately equal distance from the visual, tactile, and auditory projection areas, which Gonzalo termed *marginal* areas in this context. In the summer of 1938, several months after M was wounded, Gonzalo observed that the patient presented various visual disturbances along with some degree of agnosia, which did not seem to affect his daily life significantly. However, through careful and unbiased examinations, fundamental aspects of the functioning of sensory structures were revealed (Gonzalo, 1945, 2010, 2023).

### 3.2 Dynamic action phenomena: the central syndrome

Almost by chance, Gonzalo observed that patient M perceived objects as tilted or even nearly inverted under various conditions. In 1939, he discovered in this patient what he termed *dynamic action phenomena*, which revealed changes in brain excitability resulting from the lesion. This finding paved the way for the experimental studies he subsequently conducted on patients M, T, and others over several years. These phenomena characterized what Gonzalo defined as the *central syndrome* of the cortex, so named because of the “central” location of the lesion relative to the primary sensory areas. As he explained in the first volume of his book (Gonzalo, 1945, 2010, 2023), the physiological criterion of excitability became indispensable, making a radical change in the prevailing conceptual frameworks. The famous Schneider case described by Goldstein and Gelb (1918, 1919), a soldier wounded in the First World War and studied extensively over the years, was interpreted by Gonzalo as a *central syndrome* of intermediate degree between that of patient M and that of patient T.

In what follows, a brief description is provided of the singular dynamic action phenomena: *cerebral repercussion, asynchrony*, and *facilitation*. They were first identified and studied in 1939, and published later (Gonzalo, 1945, 1950, 2010, 2023). This is the basis that later led Gonzalo to propose a system of cortical gradients (see section 3.5) as an alternative to the rigid parcellation of the cerebral cortex.

The phenomenon that directly challenged the prevailing traditional theory of localization of brain functions in the cerebral cortex is the impact on the entire cerebral cortex of a unilateral lesion confined solely to an associative area equidistant from the undamaged primary visual, tactile, and auditory areas. Gonzalo called this phenomenon *cerebral repercussion*. Upon proper examination, he found that all the functions within each sensory system were impaired, from simple elementary sensation to more complex functions, including gnosis, which was the most severely affected. Higher functions were the most altered. The impairment affected both parts of the body symmetrically despite the lesion being unilateral. In vision, for example, both visual fields were concentrically constricted (much more in M than in T), and sensitivity decreasing from the center toward the periphery. The larger the central lesion, the greater the impact. If the lesion is nearer a primary area, the alteration becomes asymmetrical, with more intense effects in the corresponding sensory system and on the contralateral side of the lesion. This pattern was termed *paracentral* syndrome. When the lesion is situated in a primary area, its effects are limited to a single sensory system and the contralateral side of the lesion, as was well known. In this context, Gonzalo referred to it as a *marginal* syndrome, which has minimal dynamic effects. As Gonzalo pointed out, neural activity is far more widespread and unified than would be expected from the anatomical and localizationist concepts generally accepted at the time.

**FIGURE 2:**
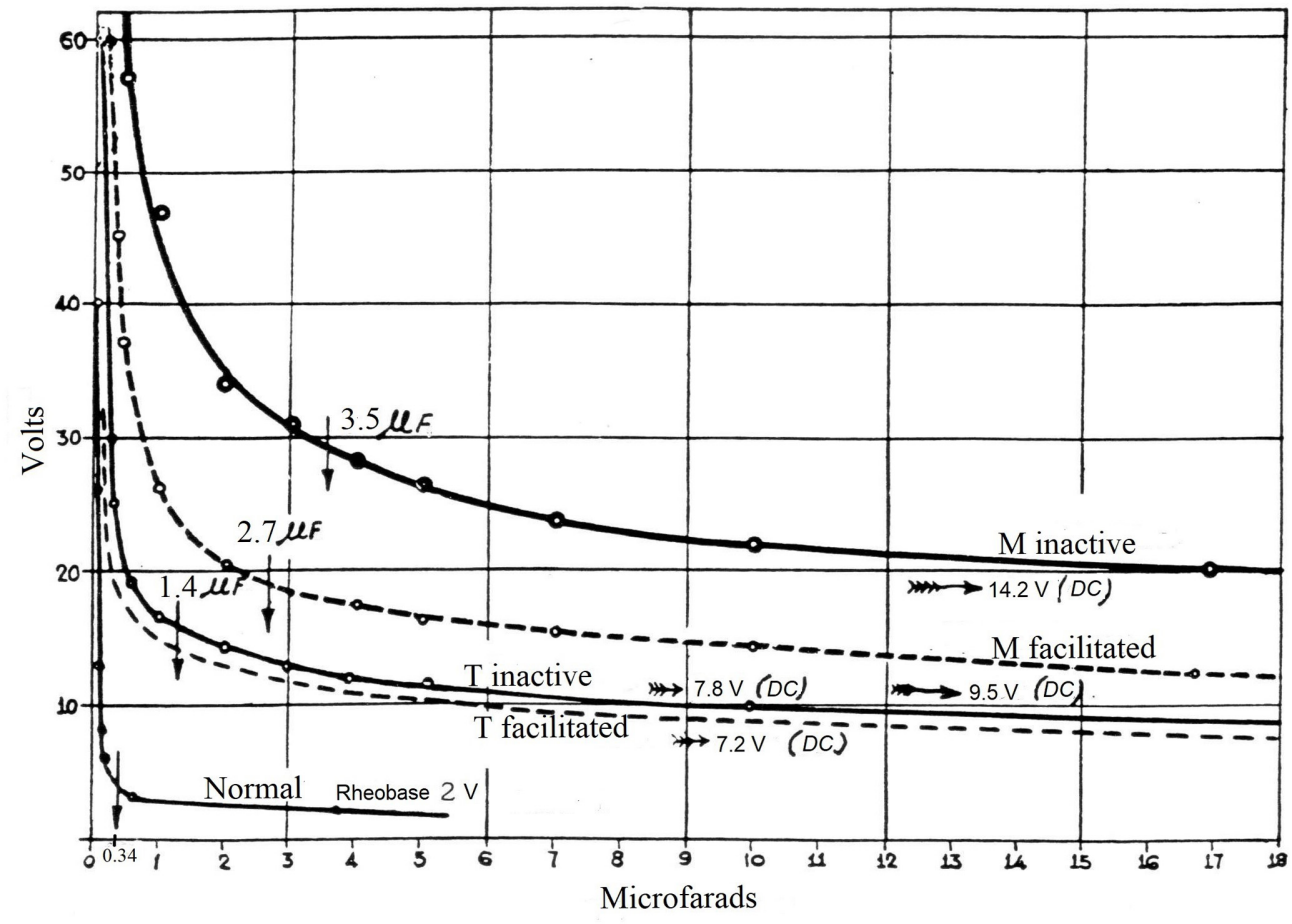
Threshold excitability strength–duration curves (volts versus microfarads) obtained by retinal stimulation using capacitor discharge (cathode on the eyelid) to elicit a minimal phosphene, for the right eye of patient M and the right eye of patient T, in the inactive state free from other stimuli (bold lines) and in the facilitated state produced by strong muscular effort (dashed lines). A normal case is included. It is worth noting the significant effect of facilitation by muscular effort in patient M and the small effect in patient T. Vertical arrows indicate chronaxie capacitances. DC, direct current. Original figure in Gonzalo (1945, 1952, 2010); English translation in Gonzalo (2023, Figure 3, p. 562). Reproduced by courtesy of RTNAC, Ediciones USC, and Editorial CSIC.

Another dynamic phenomenon is *asynchrony*, which consists of a disaggregation of stimulus perception into partial responses. In other words, perceptual phenomena that in a normal brain occur in an “all-or-nothing” manner break down into incomplete phases of perception, depending on the intensity and duration of the stimulus. Some phases involve almost inverted or diversely tilted perception in vision, touch and hearing. For example, under strong illumination, patient M perceives a white upright arrow almost correctly; however, as illumination diminishes, the arrow appears progressively greener, blurred, and tilted (rotated in the frontal plane), gradually losing its shape, decreasing in size, and finally becoming almost inverted. This process in patient T, with a smaller lesion, leads to a maximum tilt of only about 30° and less degradation of the image. Another example is the perception of a weak point stimulus on the hand. As its pressure gradually increases, up to five phases were identified in patient M, in the following sequence: a slight, non-localized sensation; a somewhat diffuse localization on the thorax; a less diffuse localization at the contralateral shoulder; a diffuse localization on the arm on the same side as the stimulus; and, finally, correct localization. Tilted or inverted perception was studied in vision, touch, and hearing for the first time in this research. Tactile and auditory inversion were discovered in examinations carried out in 1946. This topic was later reviewed in a more recent context (Gonzalo-Fonrodona, 2007). The greater the “central” lesion the greater the pathological separation between the different phases in perception. Gonzalo referred to these phases as *sensory* or *physiological levels*. More complex functions, including gnostic functions, require greater excitation and are the most altered and the first to be lost when the intensity or duration of the stimulus decreases. For several of these phases, including minimum sensation, he obtained physiological threshold curves of strength–duration type (Figure 2) in both visual and tactile functions. The observed sequence of perceptual phases led him to conclude that there is a functional continuity between elementary and higher functions and, accordingly, between primary and associative cortical areas.

Finally, the phenomenon of *facilitation* consists of an improvement in the perception of a given stimulus due to the presence of another stimulus, either of the same or a different sensory modality, or arising from the motor system. It results from neural summation that partially compensates for the deficit in excitability caused by the loss of neuronal mass in the “central” lesion. The motor system was found to act as a highly efficient facilitating stimulus, a phenomenon that remains scarcely known. For instance, the small muscular efforts required to sit or stand, as opposed to lying down, markedly enhance perception in patient M. In patient T, whose lesion is smaller, the facilitation effect is less pronounced (see Figure 2), and in healthy individuals it is barely detectable. Thus, the greater the central lesion, the greater this capability. This capability also increases when the intensity of the stimulus to be perceived decreases. However, it diminishes when the lesion is situated near a projection area. Gonzalo concluded that the “central” cortical mass constitutes a “maneuverable” and relatively unspecific structure, with the capacity for neural summation and reorganization. This provided a dynamic and integrative multisensory perspective on brain processes, well ahead of its time in relation to what is known as neuroplasticity, multisensory integration, and cross-modal effects, as indicated in the Introduction. The phenomenon of *facilitation* described by Gonzalo has been recently revisited in a contemporary context (Gonzalo-Fonrodona, 2025).

In this type of lesion, an iterative capability (temporal summation) also emerges due to the slow response of the cerebral system. As before, this capability increases as the excitability deficit caused by the neural mass lost in the central lesion becomes greater, and decreases as the lesion approaches a projection area. As Gonzalo (1945, 2010, 2023) noted, until these phenomena of facilitation and temporal summation were well understood, it was not possible to perform accurate clinical examinations. The tests required that the patient be in a state free from any stimulus other than the test stimulus and from any muscular effort, and that the measurements be performed under strict control of the intensity and duration of the stimulus, starting from threshold values. In successive measurements, stimuli had to be spaced appropriately to prevent iteration and minimize the patients’ characteristic fatigue.

**FIGURE 3:**
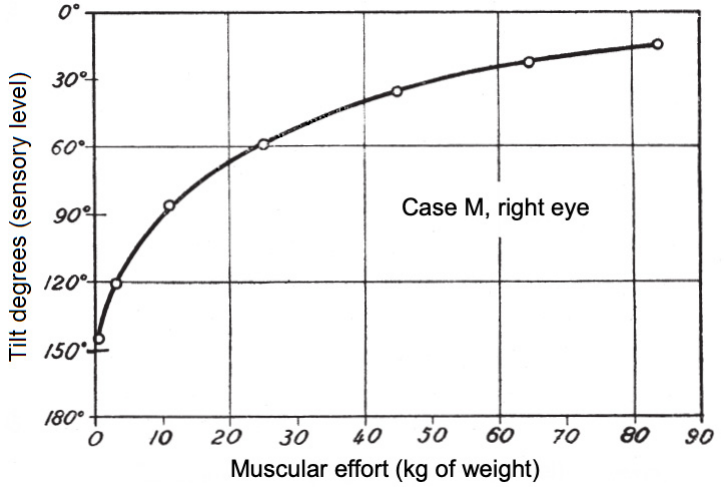
Perceived orientation (in degrees) of a 10-cm upright white test arrow as a function of muscular effort, for the right eye of patient M, 40 cm from the arrow, and a fixed luminous intensity of the arrow such that M in an inactive state perceives it as rotated 150°. As the muscular effort increases due to the weights held by the patient (indicated on the horizontal axis), the orientation of the image improves until it is almost correct, although it does not reach 0° (normal). Original figure in Gonzalo (1945, 2010); English translation in Gonzalo (2023), Figure 13.14 on p. 210. Reproduced by courtesy of RTNAC, Ediciones USC, and Editorial CSIC.

Gonzalo determined recruitment (integration) curves in patients M and T by measuring, for example, the straightening of the visual image, or the enlargement of the visual field, as the stimulus intensity increased, or alternatively, as the intensity of the facilitating stimulus increased (Figure 3). As he remarked, these curves follow Fechner’s law quite well. More recent analysis of his data show that they can be well described by power laws (Stevens’ law), which are characteristic of neural networks (Gonzalo-Fonrodona and Porras, 2007, 2009). These recruitment curves describe what Gonzalo termed *sensory growth*, whereby the patient reaches a higher *sensory level* with reduced pathological *asynchrony* (functional disaggregation).

### 3.3 Publication of *Brain Dynamics*

When the war ended and Gonzalo was able to leave Valencia, he returned to Madrid, where he was placed under surveillance by a military court for having been in the Republican zone, but he was finally acquitted of all charges in October 1940. He presented his first observations and research to the Consejo Superior de Investigaciones Científicas (CSIC) (Spanish National Research Council) in 1941 in a memory entitled *Investigaciones sobre dinámica cerebral. La acción dinámica en el sistema nervioso. Estructuras sensoriales por sincronización cerebral* (Research on brain dynamics. Dynamic action in the nervous system. Sensory structures through brain synchronization) that was awarded by this institution. In 1942, he presented to the CSIC another innovative report entitled *Investigaciones sobre el analizador cromático* (Research on the chromatic analyzer), which addressed the brain physiology involved in the formation of color sensations. Although it was selected for the national science prize awarded by the dictator Francisco Franco, the prize was declared vacant. Within the highly precarious conditions of the postwar period, he continued to study patients M, T, and others, with the support of the Cajal Institute, although, as he states in his book (Gonzalo, 1945, 2010, 2023), the publication was delayed due to the difficulty of obtaining the indispensable instruments for experimentation. However, he never considered leaving Spain, where he could be close to the wounded. He even had to go to the prison in Granada (Spain) to examine a patient. In 1942, he was appointed head of the Brain Research Department at the Cajal Institute, which was already part of the CSIC. In 1945, he became head of the Brain Pathophysiology Section.

Finally, the first volume of his book on Brain Dynamics was published by the Cajal Institute in 1945 (Gonzalo, 1945, 2010, 2023). It incorporates, to some extent, the content of the works presented to the CSIC in 1941 and 1942. In this volume, Gonzalo presents general aspects and meaning of the research conducted since 1938, and a quantitative analysis of the sensory dynamics of visual functions, from elementary excitability to schema function. He describes in great detail the variously tilted or inverted vision, the delocalization and alteration of colors, the perception of movement, its inversion, visual forms, visual agnosia, etc. Concerning schema, we mention as an example of novel finding, the striking phenomenon of what he termed *orthogonal disorder*, according to which patients M and T were able to read a text with equal ease whether it was in the normal position or upside down, without noticing the change in position. Another peculiar phenomenon is the disorder whereby allocentric orientation is replaced by egocentric orientation due to a change in spatial references. All these anomalies disappeared when patients were under facilitation, such as strong muscular effort.

The second volume had to wait until 1950 to be published (Gonzalo, 1950, 2010, 2023), also due to a lack of resources for experimentation. Here, Gonzalo describes the sensory dynamics of tactile functions such as general excitability, tactile sensations, tactile inversion, body schema and tactile gnosis. Inverted perception is thus generalized in the *central syndrome* to all sensory systems of a spatial nature, once confirmed in the auditory system. There were no precedents for this kind of tactile or auditory inversion, nor are there any today. As Gonzalo states, this type of tactile inversion, which is more difficult to detect than visual inversion, must be distinguished from tactile allochiria. Tactile inversion is studied for both cutaneous and joint stimulation, including complex processes such as walking. The body schema is considered in varying degrees according to the somatic, postural and praxis model. In this volume, Gonzalo introduces several new concepts: the *tactile field*, in analogy with the *visual field*; the *sensory field* (in a general sense) and its *dimensions* (intensity, space, and time); the *residual field* (a sensory field not fully developed), where both proximal deviation (a shift toward the center of the *field*) and inversion are involved in the spatial localization of a stimulus in *central syndrome*; and the *spiral development* of the sensory field toward normality, in which the stimulus localization follows a relatively open spiral as the intensity of the stimulus increases, or through facilitation or iteration (Gonzalo, 1950, 2010, 2023). As in vision, a continuity is also found between elementary sensory functions and higher functions such as gnosis, all based on the same physiological laws.

### 3.4 Reception of the book and the beginning of teaching activity

Internationally, the book was remarkably well received, even though it was written in Spanish. For example, we reproduce excerpts from several references to it. Particularly noteworthy is the extensive review published in the journal edited by Buscaino (Viembi, 1946), which includes remarks on the first volume such as (pp. 368–370): “T*he book is very rich in objective observations, most of them original and of great interest. It is also rich in theoretical deductions… A series of very interesting and important facts… Particularly noteworthy is the phenomenon of tilted or inverted vision, this being the first case in the international literature of almost chronic duration…*” (translated from Italian). Shortly thereafter, Bender and Teuber (1948), in a study on visual functions, stated (p. 171): “*Thus far, the American and English literature has failed to produce a monograph similar in scope to Gonzalo’s Dinámica Cerebral which was based on experiments with brain injured casualties of the Spanish Civil War.”* de Ajuriaguerra and Hécaen (1949) made numerous observations, among which the following general remark (p. 279): “…*Let us also mention, in Spanish, the very important volume by J. Gonzalo*” (translated from French). Guiraud (1950) devoted several pages to the first volume of Gonzalo’s work, and Critchley (1953) also discussed some of his findings.

Gonzalo received letters from other notable authors. In 1945, Piéron wrote^1^ : “*I am very interested in your ideas and in the facts you have brought to light…*” (translated from French). In 1946, the neurologist Bing praised Gonzalo’s work, writing^2^ : “*It is a work of the utmost importance and originality, worthy of the traditions established by the immortal sage* (Ramón y Cajal) *to whose memory it has been dedicated*” (translated from French). Köhler, one of the leading figures in Gestalt theory, wrote to Gonzalo in 1946: “*I have read it* (the book) *with the greatest interest. It contains many observations which are both new and very important. I also believe that at several points your interpretations are more convincing than those of Gelb and Goldstein*,” and regarding the second volume, he wrote to him in 1951: “…*your research has become even more important to me*” (translated from Germany).^3^

From the Spanish scientific literature, we include some translated comments. In an extensive review of the book, Germain (1945) stated (pp. 126, 137): “…*phenomena* (observed and interpreted by Gonzalo) *that in many respects revolutionize current conceptions of physiology, psychology, and brain pathology*… *it is the most important experimental biological work in many years and, in terms of technique and experimentation, it is a demonstration of genius and precision that is seldom seen in such extensive works*.” In the same year, Piga Pascual (1945) wrote (p. 260): “*Brain dynamics is destined to be the basis for very important experimental work and to resolve clinical issues of utmost interest*.” In a summary of the first volume, Tolosa Colomer (1945–1946) remarked (p. 815): “…*Gonzalo’s contributions go far beyond the limits that could be established in a clinical or pathophysiological study of wounds in the parieto-occipital cortex.*” From the article by Germain (1946), in which Gonzalo’s research is analyzed in relation to other authors, we include the following excerpt (p. 428): “… *from the war we had in Spain emerges Gonzalo’s work, which we are going to examine again (…) and which has already earned praise from Professors Köhler, Bing, Piéron, Lashley, and Katz, among others*. …*Gonzalo, a fine-minded neurologist (…) has moved beyond the localizationist concept, shedding preconceived ideas with an uncommon experimental spirit, and steering research along uncharted paths.*” In a medical treatise, Pedro-Pons (1952) commented on the topic of agnosia (p. 153), noting that “*Gonzalo reviews the problem in a revolutionary way.*”

Lafora, then exiled in Mexico, wrote two letters to Gonzalo in 1946.^4^ In the first, he requested a summary of the volume and added: “… *I would not want to fail to praise the work of a Spaniard who holds our name in such high esteem.*” In the second letter, written the same year, he stated: “…*I find your book excellent, returning to the principles of physiology so often forgotten by clinicians…*” (both excerpts translated from Spanish). In relation to the second volume its author was awarded a prize by the Royal Academy of Medicine of Spain in 1951, and he received more comments, including one from Jiménez Díaz in a 1950 letter,^5^ who described the work as “*marked by originality and scientific rigor*” (translated from Spanish). That same year, Tolosa Colomer wrote^6^ : “*Once again, I am amazed by the effort and skill involved in publishing a work of this kind, which revisits the most difficult and abstruse problems in neurology and the physiology of the senses… I must repeat that it will be very difficult to evaluate your research work in Spain*” (translated from Spanish).

The first volume sold out quickly but was not reprinted, despite requests from its author in 1953, who had prepared new illustrations for that purpose. It is therefore easy to imagine the impossibility of carrying out a translation of the volume.

**FIGURE 4:**
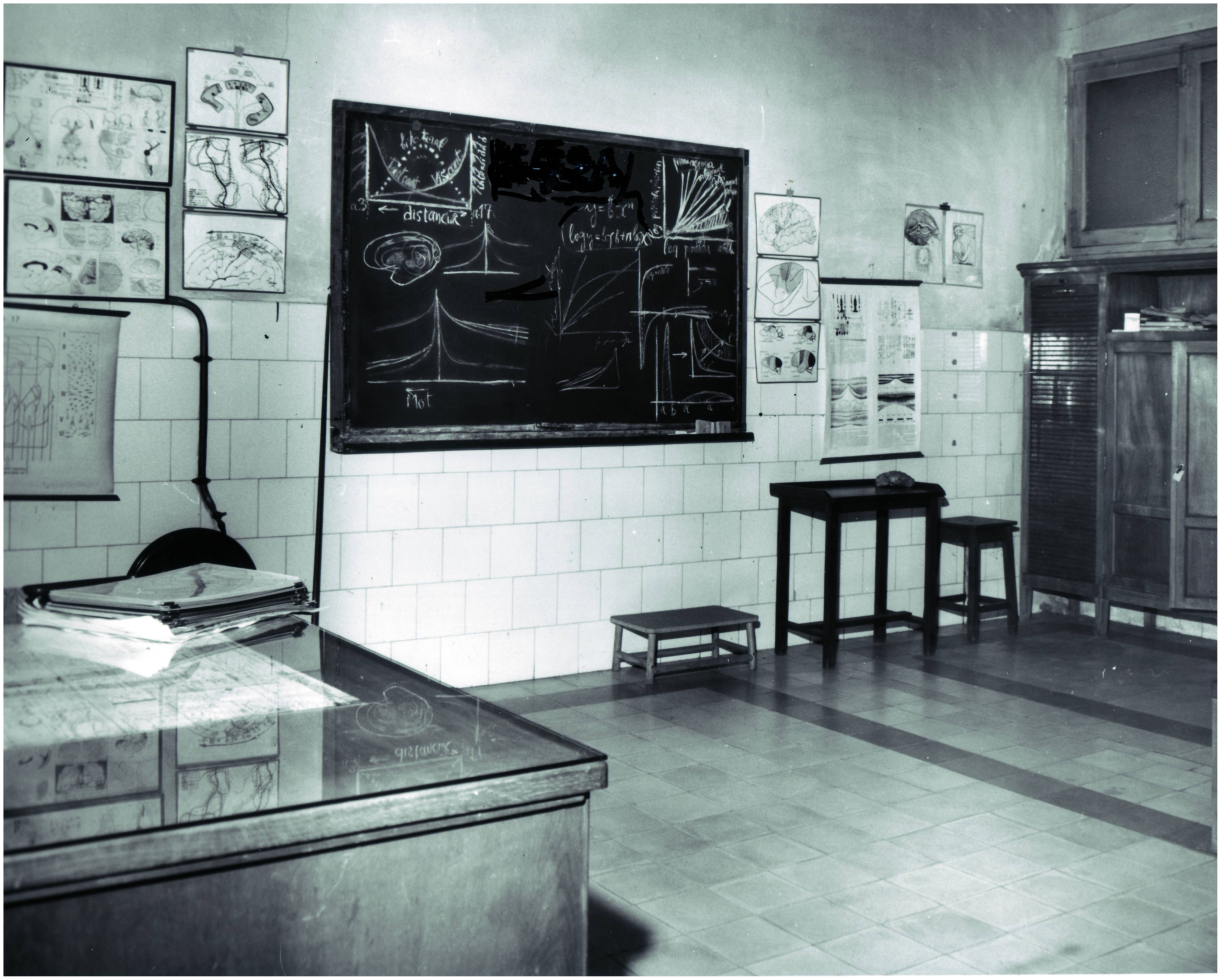
Room where Gonzalo taught the courses on Brain Pathophysiology (1945–1966), which formed part of the laboratory of the same name, where he examined brain-injured patients. It was located in the former Faculty of Medicine (Calle Atocha 106), part of what was then known as the “Universidad Central de Madrid.” On the blackboard are drawings made by Gonzalo explaining cortical gradients. Justo Gonzalo Archive. Published in Gonzalo-Fonrodona (2024, Figure 1) and reproduced here with the permission of Ediciones Doce Calles.

In 1945, the then-called “Universidad Central de Madrid” approved Gonzalo’s proposal to contribute to doctoral education by offering a course on brain pathophysiology. He was appointed the professor in charge for the course, which he taught for 21 consecutive academic years, starting in 1945–46. The course consisted of three weekly sessions over a 7-month period. Initially, 22 students enrolled, but by the 1949–50 academic year, the number had grown to 71. Financial compensation was minimal, and during the first 2 years, Gonzalo received no remuneration. In his lectures, Gonzalo presented his research in detail. He conducted these courses in the former Faculty of Medicine building (Figure 4), where he also directed the Laboratory of Brain Pathophysiology, affiliated with the Spanish National Research Council (CSIC), in particular with the Cajal Institute. There, he continued his clinical investigations studying patients M, T, and others, some of whom had been referred by the neurosurgeons Obrador and Tolosa, as well as the psychiatrist Montserrat and the anatomical pathologist Hernando de Larramendi, among others.

### 3.5 New data, secondary cortical areas, and cortical gradients

The 1950s were a highly fruitful period in which Gonzalo obtained new clinical data and introduced novel concepts. His 1951 publication (Gonzalo, 1951) remained incomplete, but in a later publication (Gonzalo, 1952, 2010, 2023 Suppl. I), Gonzalo reported additional cases of *central* and *paracentral* syndromes and developed new conceptual frameworks based on his previous research, with implications for the organization of cortical neural centers. One of these conceptual frameworks is what he called the *spiral development of the sensory field* in the brain’s integrative process, which involves a re-inversion of the inverted image formed on the retina. It was partially explained in the second volume of his book, but in this publication, he takes into account both Cajal’s theory of nerve fiber crossings (Ramón y Cajal, 1899) and the secondary visual areas discovered shortly before. He argued that the secondary areas, by reinverting the image and rendering it bilateral, eliminate any spatial incongruities, and proposed that the pathological process of inverted or tilted perception in his patients would be caused by an asynchrony between the primary and secondary areas of the cortex.

**FIGURE 5:**
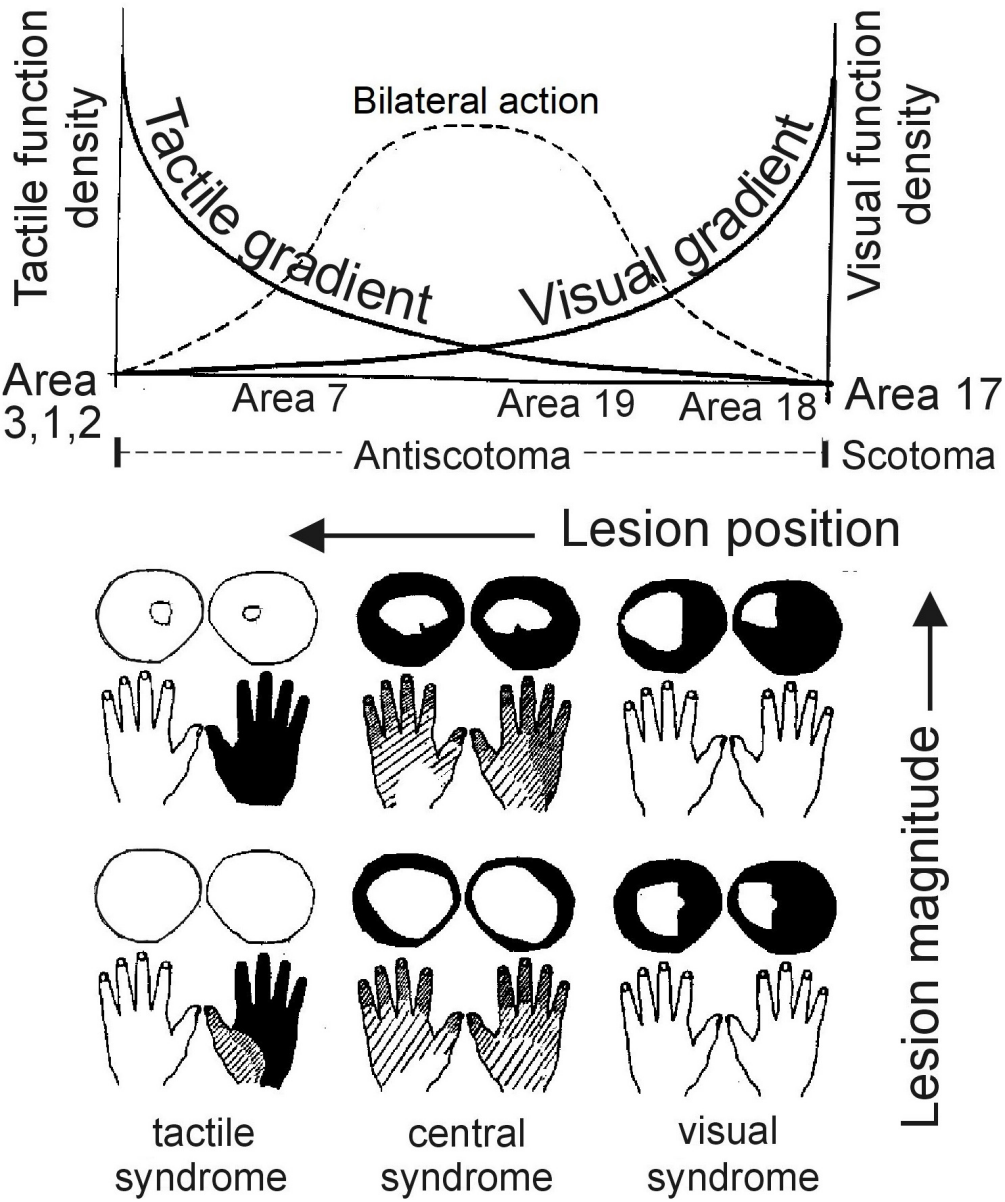
Upper part: Schematic diagram of the specific visual and tactile gradients. The curves that take their maximum value in the visual and tactile projection areas represent, respectively, the densities of visual function and tactile function, spreading in gradation across the cortex. They have a contralateral action. The central bell-shaped curve represents the bilateral action (via the corpus callosum) and the multisensoriality that results from the superposition of the specific gradients, with a maximum in the “central” region. Bottom: Cases studied by Gonzalo arranged according to the position of the cortical lesion (indicated on the horizontal axis of the upper diagram) and the magnitude of the lesion as indicated by the vertical arrow. The most affected parts of the visual fields and hands are darker. The cases with *central syndrome* are in the middle; the others present visual (right) or tactile (left) paracentral syndromes. Figure adapted from Gonzalo and Gonzalo (1996, p. 84, Figure 5), with permission of The MIT Press. ©© 1995 Massachusetts Institute of Technology. All rights reserved. Similar figure in: (Gonzalo, 2010, 2023, Suppl. II, Figure 6).

In the same 1952 publication, Gonzalo presented one of the culminations of his previous research, namely the concept of cortical gradient along the cortex, as an alternative to the traditional rigid anatomical parcellation of the cortex into different areas for the localization of brain functions. According to Gonzalo, a specific cortical gradient expresses that the specificity of a function (e.g., visual) would be distributed gradually throughout the cerebral cortex, with a maximum density value at the corresponding projection area (e.g., visual) and decreasing across the cortex until reaching other primary areas, as shown in the simplified diagram of the specific visual and tactile gradients in Figure 5. This type of gradient has a contralateral action, and responds to several observed facts. One is the significant involvement of the extravisual cortex (parieto-occipital, parietal, temporal, etc.) in the visual field; the same can be said for the tactile field. Another is that the effect of a cortical lesion depends not only on the position of the lesion, but also on its magnitude; thus, a visual deficit produced by a small lesion near the primary area requires an extensive lesion in the “central” region. This region, where specific gradients overlap to the maximum extent (see Figure 5), would be a rather multisensory (or nonspecific) area, with a maximum bilateral effect due to the corpus callosum, from which projection areas are excluded, as is well known. It is expressed in Figure 5 by the bell-shaped curve that takes maximum values in the “central” region. This gradient system is an expression of the gradation observed between the different cortical syndromes: *central*, *paracentral*, and *marginal* (of the projection area). Gonzalo uses, an element of spatial nature to compare the syndromes with each other: the sensory field. For example, the shape and size of the visual field vary from one syndrome to another according to defined rules, as shown in Figure 5.

Unlike rigid separation into areas, the proposed macroscale cortical gradients offer functional continuity with regional variation: each point of the cerebral cortex participates differently in both the contralateral specificity of the specific gradient and in the bilateral central action of a rather multisensory or nonspecific nature. Each point thus acquires different properties from those surrounding it, while maintaining a certain unity with the rest. In Gonzalo’s words, functional localization is refined at each point of the cortex; the sensory field projected onto the primary area is only a sketch that must be developed (integrated) toward the “central” region throughout the cortex. Thus, for the visual field to be normal, with acuity 1, etc., the action of the entire visual gradient across the cortex must be involved. These gradients represent the dynamic aspect of their anatomic basis (terminal paths, primary and secondary areas, decussations, corpus callosum, etc.).

Gonzalo later proposed, based on data from other authors, what he called complex gradients, such as a mixture of tactile and motor gradients: a double specific gradient with a maximum at the Rolandic fissure and decreasing both gradients toward the frontal pole with higher values of the motor gradient, and also decreasing toward the parietal region but in this case with higher values of the tactile gradient. He also extended the concept of gradient to language in relation to different types of aphasia. All of this is collected in a posthumous publication (Gonzalo, 2010, 2023, Suppl. II).

Decades later, a similar concept of cortical gradients was proposed in humans (Goldberg, 1989). Cortical gradients are now identified using neuroimaging techniques and are considered fundamental to the organization of the brain, as mentioned in the Introduction along with relevant references.

**FIGURE 6:**
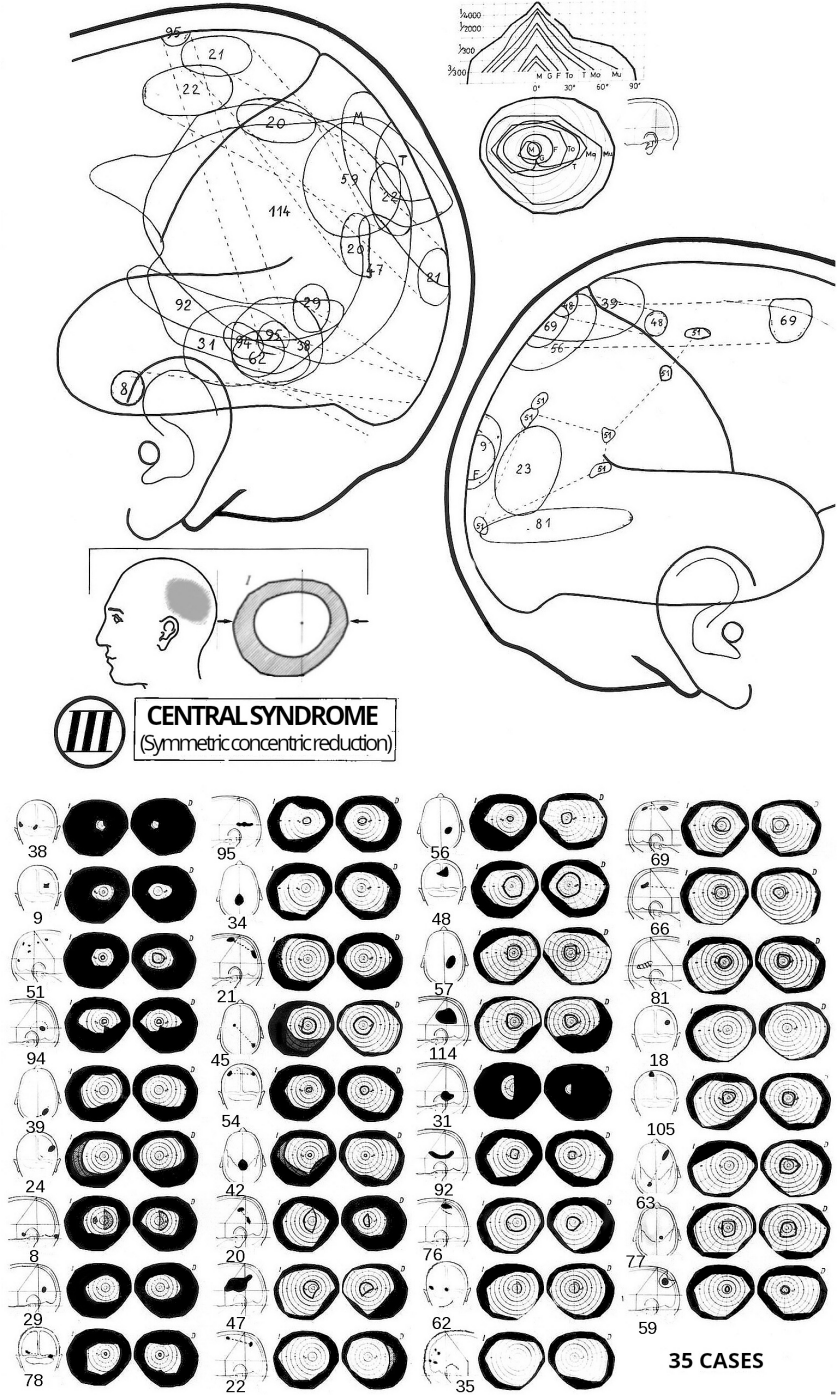
Thirty-five cases of *central syndrome* of varying intensity. The symmetric concentric reduction of the visual fields is shown, with numbers identifying the individual cases. Upper part of the figure: the areas of the corresponding lesions in both the right and left hemispheres. A small schematic diagram illustrates, in a general way, the lesion area for a single hemisphere and the type of visual field alteration. At the top of the figure, visual sensitivity profiles for different cases with varying degrees of concentric reduction are shown. Original published in Gonzalo (2010, Supl. II, Figure 3). English translation in Gonzalo (2023, Supp. II, Figure 3). Reproduced by courtesy of RTNAC, Ediciones USC, and Editorial CSIC.

In 1951, Gonzalo completed an exhaustive search throughout Spain, begun in 1946, for people with war-related brain injuries. From the more than 2.500 cases listed in the Register of War Wounded for the Fatherland, which excluded those on the defeated side, he selected approximately 200 subjects (Gonzalo-Fonrodona, 2024). Throughout 1952 and 1953, Gonzalo examined these individuals from different parts of Spain at the Laboratory of Brain Pathophysiology at the Faculty of Medicine in Madrid. He also re-examined several patients he had first seen in Valencia during the war, including patients M and T. This research was also funded by the Cajal Institute, and involved the collaboration of the doctors César Paumard and Rafael Thomas, who had excelled in Gonzalo’s doctoral course. Based on these examinations, Gonzalo documented 35 cases of *central syndrome* (see Figure 6), along with an equal number of cases of *paracentral syndrome*. During these years, he continued examining additional patients, many of whom were referred by Obrador, Lafora, Díaz Gómez, Hernando de Larramendi, Marañón, Martín-Santos, Varela de Seijas, among others.

**FIGURE 7:**
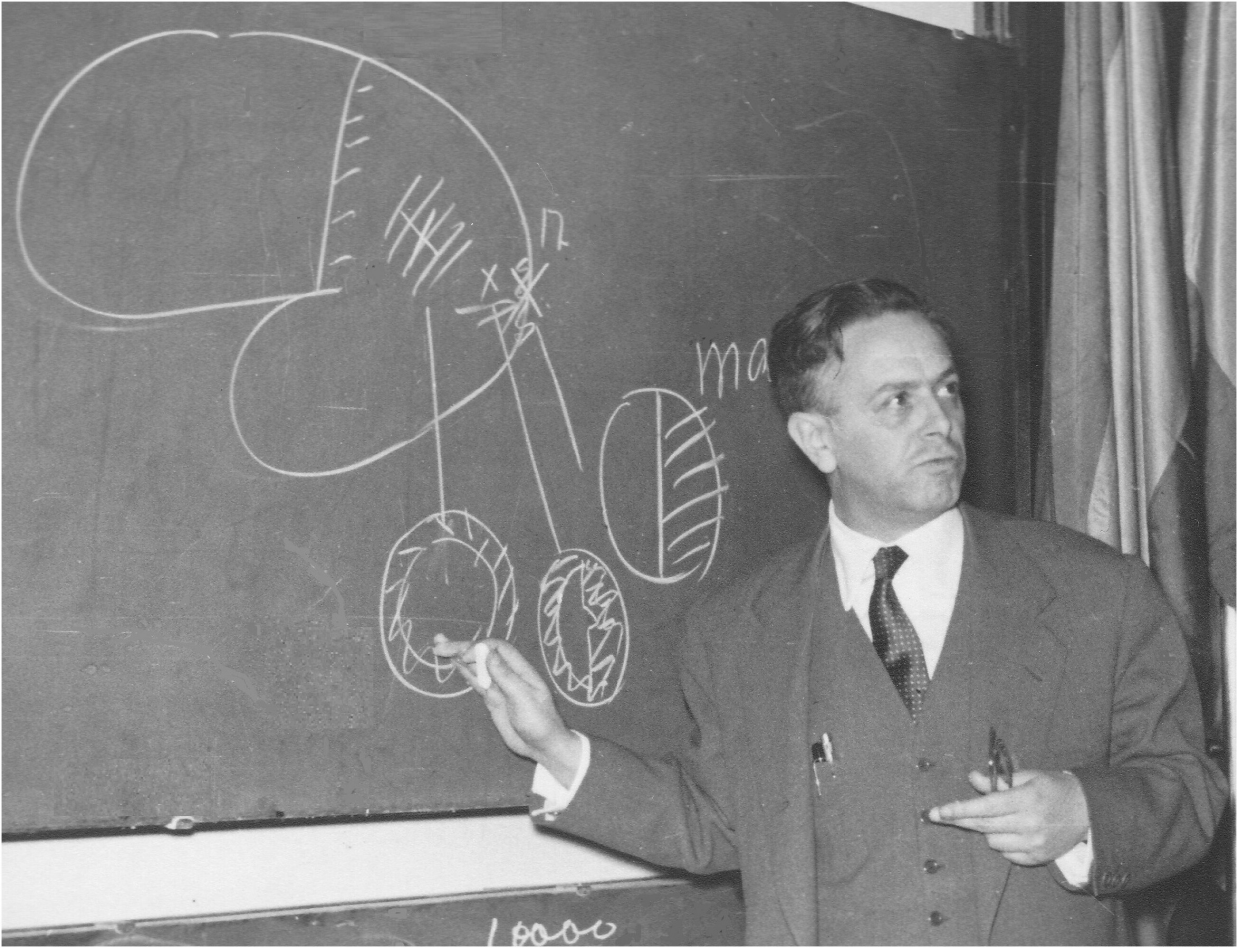
Justo Gonzalo presenting his contribution at the IV Congress of Neuropsychiatry in Madrid, 1954. The blackboard shows a diagram of the brain and three visual fields altered according to the *central*, *paracentral*, and *marginal* syndromes, depending on the location of the cerebral lesion. Published in Gonzalo-Fonrodona (2024, Figure 3) and reproduced here with the permission of Ediciones Doce Calles.

In 1954, Gonzalo presented the contents of his 1952 publication (Gonzalo, 1952, 2010, 2023 Suppl. I) at the IV Congress of Neuropsychiatry, held in Madrid (Figure 7), and received notable comments from leading figures in the field. Translated from Spanish, some of these are: “*We should mention the presentation by Dr. Justo Gonzalo, which was full of originality and numerous discoveries and innovations”* (Rodríguez Lafora, 1954, p. 283); “…*interesting presentation that of Gonzalo, who with such perseverance and honesty has been carrying out his meticulous observations and studies for so many years*” (Obrador, 1954, p. 280); “*I thought your presentation was wonderful… I presume this opinion must be shared by everyone. It is refreshing to see a Spanish work created with a spirit of observation and originality…*” (letter from Marañón to Gonzalo);^7^ and “*The best part of the congress for me was listening to you… Your work and your way of researching have been a revelation and an example to follow*” (letter from Castilla del Pino to Gonzalo).^8^ Also noteworthy is the publication by Roldán Viller (1955), which provides a summary and analysis of Gonzalo’s studies, from which we quote (p. 231): “…*despite the relative silence that has generally surrounded the publication of these studies, we consider them to be of great theoretical importance*” (translated from Spanish).

Gonzalo did not participate in any other conferences or congresses, despite receiving several invitations, stating that he needed to devote all his time to research, an activity he never interrupted, except in cases of serious health problems.

### 3.6 Allometry of brain functions in a “central” lesion

Another concept that Gonzalo introduced and explained in his doctoral courses around 1952 was the concept of *allometry* in relation to the different (allometric) changes that different sensory functions undergo in a “central” lesion. This concept, closely related to that of *dynamic similarity*, is inherent to dynamic systems, including biological systems, and governs their growth processes. Here it is applied to the sensory dynamics of the cerebral cortex.

Gonzalo observed that in *central syndrome*, once a new balance of the brain system was established, the physiological intensity-duration curves of the various functions, the sensitivity profile of the concentrically reduced visual field, and other functions, retained the same shape as in healthy subjects, but with reduced values depending on the magnitude of the “central” lesion. This led him to assume that central syndromes of different intensities show a dynamic similarity with the normal brain and with each other, that is, they have the same organizational plan, but on a reduced scale (Gonzalo, 1945, 1952, 2010, 2023). It is known that under a change of scale, the different parts of a dynamic system change differently, i.e., allometrically, and relate to each other according to scaling power laws. This applies, for example, to the growth of organisms. Gonzalo applied it to the “reduction” of the cerebral system due to the “central” lesion, and quantitatively analyzed the change observed in different functions. He found a relationship, expressed graphically by a curve, between the tilt (orientation) of the visual image and the width of the visual field in 24 cases of lesions of different intensity (Gonzalo, 1952, 2010, 2023, Suppl. I).

**FIGURE 8:**
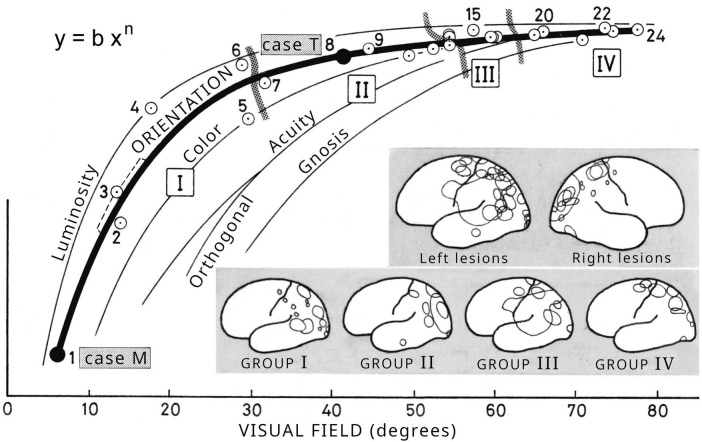
Allometry. Correlation curves of various visual perceptual functions (luminosity, image orientation, color, visual acuity, and gnosis) with the size of the visual field in 24 cases examined by Gonzalo (see the text). The cases are classified into four groups, from most affected (Group I) to least affected (Group IV). Original figure in Gonzalo (2010, Supl. II, Figure 19); English translation in Gonzalo (2023, Supp. II, Figure 19). Reproduced by courtesy of RTNAC, Ediciones USC, and Editorial CSIC.

In 1956 he added to this curve other curves for luminosity, color, acuity and gnosis, which show different (allometric) relationships with the visual field width (Figure 8). This expansion of concepts is included in a posthumous publication (Gonzalo, 2010, 2023 Supp. II). These curves approximately follow power laws of the type *y* = *b x^n^*, where *y* denotes different functions (acuity, visual orientation, color, etc.), *x* is related to the size of the visual field, *n* (positive number less than unity) is the allometric coefficient, which is different for each function, and *b* is a constant. A practically normal sensory level (normal brain) corresponds to the point at which the functions have the highest possible value and are united (case 24 in Figure 8). In patient T (case 8 in the figure), all functions decrease, but differently; the most affected is gnosis, followed by visual acuity, color, etc., and finally simple luminosity. This order is always constant. In the most severe case of patient M (case 1 in Figure 8), several functions have already been lost in the inactive state, the orientation of the image is almost inverted but still retains some luminosity. Thus, the graph of Figure 8 illustrates the disaggregation (or asynchrony) of functions in the pathological cases. These curves are of the same type as those observed in the *sensory growth* of a single individual, such as patient M, when he moves from a low to a higher *sensory level* by increasing brain excitability through greater stimulus intensity or facilitation. It is therefore understandable that a case such as that of patient M, with a large separation between his partial functions, makes it possible to study the organization of the cortical sensory structures.

This generally unknown topic of sensory allometry has been addressed in publications based on Gonzalo’s data, where the corresponding exponents of several power functions have been derived (Gonzalo, 1997; Gonzalo-Fonrodona, 2009; Gonzalo-Fonrodona and Porras, 2014).

In 1958, Gonzalo was awarded a prize by the Spanish Psychological Association. References to his research continued to appear. An example is Cabaleiro Goas (1959), who summarized Gonzalo’s work and stated (p. 623): “… *the dynamic theory of this Spanish researcher is highly fruitful, especially in the field of neurology and specifically in relation to sensory functions, its extraordinary value being recognized in the world of neurophysiology*” (translated from Spanish).

By the late 1950s, Gonzalo had compiled an extensive body of clinical data from his own practice and from relevant authors abroad, while also developing new theoretical concepts. He had long been planning a new volume on brain dynamics, as he rejected the idea of publishing his research in a fragmentary manner. Instead, he gladly explained all the details of his work in the annual doctoral courses on brain pathophysiology, including unpublished material, such as the concepts of complex cortical gradients, similarity and allometry. This is documented in the class notes and coursework produced by his doctoral students.^9^ It is clear that he did not teach these courses for financial gain, as he received only a symbolic remuneration. The courses attracted an increasing number of students who followed them with great interest. Some of them made this research known in their countries of origin, or in the countries to which they emigrated due to the lack of opportunities in Spain. This was the case of Hernando de Larramendi at the Rockefeller Institute in New York in 1957.^10^

Gonzalo occasionally consulted former students for their opinions on the course, particularly regarding comprehension, content, and methodology, and also invited them to offer suggestions. Below are brief excerpts from some of these opinions, translated from Spanish^11^ : “*The theory of Brain Dynamics is original, harmonious, and easily understandable, with a fully structured biophysical view of the brain.*” (A. Martínez, course 1957–58); “*What struck me most was the originality and scientific approach of the course.*” (D. Roca, course 1959–60); “*The theory of Brain Dynamics is truly doctoral-level, presented in an engaging and understandable way despite its complexity. This is demonstrated by the regular attendance of interested students despite the lack of hospitality offered by the laboratory where it is currently explained.*” (M. García, course 1963–64); “*The concepts of gradient and allometry are so suggestive and attractive, and are supported by so much data, that they should be emphasized.*” (J. C. Legido, course 1964–65); “*I found the presentation of the new theory clear, understandable, and appealing… It caught my attention, from the most basic aspects to the allometric arrangement of sensory functions.*” (M. T. García, course 1965–66).

### 3.7 Setbacks, extension of concepts, and application to cybernetic brain models

Gonzalo maintained contact with patients M and T, regularly asking them about their health and their precarious financial situation. He was deeply moved to learn that patient T had died in 1960 from a digestive disease.

References to Gonzalo’s research continued to appear (Rodríguez Arias, 1961; Pellegrin et al., 1961; Ballús, 1964). We quote only the comment by Cabaleiro Goas (1966, p. 281): “…*In contrast to localizationist concepts that viewed the brain as a mosaic of functions, such as those held by Wernicke or Kleist, other fully dynamic conceptions have emerged, such as those of Lashley, Goldstein, or Gonzalo*” (translated from Spanish).

By 1963, Gonzalo already held the position of Scientific Researcher at the CSIC, initially at the Cajal Institute, and from 1969 onwards, at what was then known as the “Patronato Alfonso el Sabio,” where he headed the Brain Pathophysiology Section. He worked mainly alone and without interruption, with the help of some former doctoral students for specific problems and always with the support of his wife, Ana María Fonrodona since 1945. The draftsmen’s work was particularly intense, as they drew the numerous illustrations that Gonzalo had designed. He did not attend meetings, seek praise, or act for financial gain. His interest was mainly focused on his research. Throughout his life, his intellectual curiosity extended far beyond this, developing a deep interest in physics, mathematics, philosophy, biology, and the arts.

Administrative problems worsened in 1966 when the Dean of the Faculty of Medicine sent Gonzalo a formal notification indicating that the course could only be maintained if it were sponsored by a department with which he had no connection.^12^ Gonzalo sought to preserve his academic independence, and the course was ultimately discontinued. In vain was the formal request addressed to the Dean by several professors to continue the course. In this document, after praising Gonzalo’s teaching and research work, the last paragraph translated from Spanish reads^13^ : *“…for the benefit of teaching and research…. we believe it is appropriate to facilitate the resumption of this course on pathophysiology of the brain. It is the least we can do to show our gratitude for the selfless and effective work of Dr. Justo Gonzalo.”*

From then on, there were more administrative setbacks and also health problems. The following comments by Moya (1967), translated from Spanish, are worth noting (p. 9): “*M. Peraita prematurely dead, the only one dealing with neurological matters in Madrid is Justo Gonzalo, a clinician and researcher out of the common (…) who interpreted the data (…) providing an original solution, the concept of gradients, to the problem of the localization of functions in the cerebral cortex.”* Regarding the limited impact of his work, he stated: “*It is due to several factors: the great economic difficulties it faces, the indifference of most of his colleagues towards his work, his own pessimistic outlook on the future of science in Spain, and his overly critical attitude towards his own work, which has prevented him from disseminating it as it deserves.*” As for the doctoral courses, he added: “*His presence in the University as professor of a doctoral course, is -with his original, updated, sharp course- the only encouragement to neurological vocations that has been present for years and years in the Faculty of Medicine of Madrid.*”

With the relocation of the former Faculty of Medicine to the university campus, Gonzalo also faced insurmountable obstacles in transferring the Brain Pathophysiology Laboratory, which was ultimately closed in 1970. However, this did not prevent Gonzalo from continuing to develop his model, extending the gradient system to language and types of aphasia, and progressing toward what he called a *neurophysics* of the cerebral cortex, as described in a posthumous publication (Gonzalo, 2010, 2023, Supp. II).

References to his research continued to appear (Barraquer Bordas, 1968; Ballús, 1970; Barraquer Bordas, 1974; Siguán, 1976), even dedicating an entire chapter to it (Llopis, 1970). It is worth mentioning the extensive summary of Gonzalo’s research that includes brain gradients and allometry, from which we highlight the following excerpt (Roldán Viller, 1975, pp. 45, 46): “*…the author who, through his personal research, has developed a new theory on brain localization is Dr. Justo Gonzalo, who, with Germanic tenacity and a profound scientific spirit, has demonstrated the existence of the central syndrome….Dr. Gonzalo, less understood in Spain than abroad, began by meticulously studying all sensory aspects in two patients with brain injuries caused by our war…. He now has more than 100 brain injury cases that he has personally studied”* (translated from Spanish).

In 1976, physicists and engineers interested in cybernetic models of the brain contacted Gonzalo. The result was a Ph.D thesis entitled *Modelos Cibernéticos de Dinámica Cerebral* (Cybernetic Models of Brain Dynamics) (Delgado, 1978), which includes the following dedication translated from Spanish: “*To Dr. Justo Gonzalo, who coined the term ‘brain dynamics’ and whose studies on sensory aphasia point to an important avenue for quantifying cortical functions.*” We also highlight some quotes from the thesis, translated from Spanish: “*Here we attempt to relate the organization of neural tissue to behavior. The three main researchers in this field are Lashley, Luria, and J. Gonzalo”* (p. III), “…*J. Gonzalo’s gradient theory encompasses Lashley’s law of mass action as a special case and is more accurate than Luria’s*” (pp. IV and 29).

### 3.8 Last years and beyond

During the 1970s and early 1980s, Gonzalo, who was already suffering from various health problems, carried out numerous illustrations related to his research. Several of them are compositions that compile data and concepts. In his final reports submitted to the Spanish National Research Council (CSIC), and reproduced in posthumous publications (Gonzalo, 2010, 2023, Supp. II), he summarized the theoretical aspect of his research as: “*Brain dynamics according to gradients, similarity, and allometry*.” More specifically, he stated: “*The brain dynamics developed…constitutes a neurophysics of the cerebral cortex. The cortex would be a system organized in gradients, which changes its metric scale in* (central) *lesions, preserving the same functional plan (functional similarity), and whose multiple particular functions change allometrically according to their respective allometric coefficients*” (Gonzalo, 2023, p. 604).

In 1983, a request to the Spanish Ministry of Education and Science to support and preserve Gonzalo’s research was unsuccessful. The same year, a reference to Gonzalo (Pérez Pérez, 1983) stated (pp. 298–299): “…*let us remember Prof. Dr. Justo Gonzalo, who, with his work ‘Brain Dynamics’, made a fundamental contribution to neuropsychology. It is regrettable, as Germain says, that Dr. Gonzalo’s work has not been sufficiently read, since the message of his work is sufficiently fruitful to be more present today*.” Later, Moya (1986, p. 136) wrote: “*One of Lafora’s most original disciples is Justo Gonzalo (…). Like all innovators, with a few honorable exceptions, Justo Gonzalo has been a victim of incomprehension and envy, both inside and outside the Faculty of Medicine in Madrid, where his courses were among the few that were complete, modern, and inspiring*.”

An exception to the general indifference surrounding Gonzalo’s research was the unconditional support of several scholars, as well as the enthusiastic reception of his work in the fields of cybernetics and artificial intelligence, which continued long after his death.

In 1985, Gonzalo issued a final medical certificate for patient M so that, under the new government law, he could be officially recognized as a war casualty and receive a pension. As a war-wounded soldier on the Republican side, patient M had not yet received any financial assistance.

Gonzalo passed away on September 28, 1986, at the age of 76, due to heart disease. After his death, references to his research continued to appear for several years among researchers in the field of artificial intelligence (Mira et al., 1987; Mira et al., 1995; Mira et al., 1996; Mira and Delgado, 2003). In this context, the *Red Temática en Tecnologías de Computación Natural/Artificial* (Thematic Network on Natural/Artificial Computing Technologies) took the initiative to publish a facsimile edition of Gonzalo’s book on brain dynamics, adding supplements (Gonzalo, 2010), where Supplement II includes the first edition of previously unpublished material from his final years of research. It is worth noting that previous requests to various institutions for the reissue of the original volumes had been unsuccessful.

Erol Başar, one of the most prominent neuroscientists of the past century in the field of brain dynamics, responded in 1991, after receiving an English translation of Gonzalo, 1952 article, as follows^14^ : “*Thank you for your confidence in sending me such an important work.*” Later, he disseminated it at the conference he organized in Lübeck, Germany, in 1994, titled “Alpha Processes: An Integrative Approach to Neuroscience.” Finally, the English translation of the 2010 facsimile edition with supplements was carried out, and the Spanish National Research Council published it in 2023 as an open-access edition (Gonzalo, 2023), thus making it readily available to the neuroscience community.

## 4 Discussion

Aspects of Gonzalo’s life and research have been presented in parallel, following an approximate chronological order. He developed research on brain dynamics under quite adverse conditions, marked first by the Spanish Civil War and later by the long postwar period in a country devastated both scientifically and economically. Gonzalo’s independent character was not always well received, and he faced serious administrative difficulties. It was unfortunate that his work in Spanish was neither republished nor translated. As a result, over time his research was practically ignored by the international community, despite its excellent initial reception. In Spain, apart from the authors who praised it, over time there was a certain tendency toward silence. It was necessary to wait for researchers in cybernetic models to appreciate the potential of his work and apply it in their research. Many years after Gonzalo’s passing, neurologist Barraquer Bordas (2005) discussed his work in a historical context and partly justified the relative silence surrounding it, stating (p. 172): “…*it must be said that his work possessed a dazzling originality that probably ‘blinded’ and alienated many*” (translated from Spanish).

As for Gonzalo’s work, it should be noted that, despite the difficulties, he carried out an extensive research that stands out for its novelty and unity. Based on clinical data and physiological principles, he developed concepts that are coherently and logically related. He characterized the *central* and *paracentral* syndromes, revealing aspects of sensory organization, the dynamic unity of the cortex, and the continuity between elementary and gnostic functions. He discovered and analyzed previously unknown pathological perceptual phenomena, and quantitatively determined relationships for both threshold perception and perception enhancement with stimulus intensity and facilitation. His research up to 1950 led him, shortly thereafter, to propose specific functional mechanisms of the cerebral cortex: first, he explained how tilted or inverted perception would obey an asynchrony between the primary and secondary areas in the integrative process; and second, he introduced the concept of cortical gradients as an alternative to rigid modular models of brain function localization, thus blurring the separation between primary and association areas, and identifying cortical regions with the greatest capacity for multisensory summation and reorganization (plasticity). According to Gonzalo, the development of what he termed the *sensory field* is an integrative process initiated in the primary area and involving the entire cerebral cortex through the gradient. This approach explained the different cortical syndromes. Gonzalo found that, in *central syndrome*, the loss of functions follows a specific sequence, with the most complex functions being the most affected, and are related to each other by allometric scaling power laws, which is consistent with the change in scale involved in the loss of “central” neuronal mass. This aspect of sensory allometry seems to be little known at present. He applied his analysis to the visual, tactile, auditory, and language systems.

From a clinical point of view, Gonzalo noted that the *central syndrome*, in more or less pure and intense forms, would occur quite frequently in cortical lesions not directly affecting a primary area. The difficulty in detecting the *central syndrome* would stem from the lack of an adequate clinical examination of patients under appropriate conditions (Gonzalo, 1945, 2010, 2023). Gonzalo also noted that the regularity of functional loss in the *central syndrome* would make it possible, on the basis of a single pathological datum, to infer deterioration in other functions. From a practical point of view, it is also worth noting the great benefit for patients to improve their perception through muscular actions, a type of facilitation little known today.

In more recent decades, several studies have mathematically formalized some of the relationships originally described by Gonzalo, such as the allometric power laws (Gonzalo, 1997; Gonzalo-Fonrodona, 2009), and the relationships between perception and stimulus intensity and facilitation (Gonzalo-Fonrodona and Porras, 2007, 2009; Gonzalo-Fonrodona, 2009), which seem to arise from universal power laws of biological growth in relation to general mechanisms of biological neural networks. A model has also been formulated for excitability threshold curves, which explains aspects of time perception in *central syndrome* (Gonzalo and Porras, 2001; Gonzalo-Fonrodona and Porras, 2015). Following Gonzalo’s criteria and methods, an improvement in Vernier visual acuity (superacuity) has been found in normal individuals under the influence of moderate muscular effort (Gonzalo-Fonrodona and Porras, 2013, 2014), in accordance with the similarity established by Gonzalo between *central syndrome* and the normal brain.

From a historical perspective, several works can be cited (Arias and Gonzalo, 2004; Barraquer Bordas, 2005; Gonzalo-Fonrodona, 2011, Gonzalo-Fonrodona, 2024, 2025; García-Molina, 2015, 2023; García-Molina and Peña-Casanova, 2022; García-Molina and Gonzalo-Fonrodona, 2023, 2024). It is worth noting the statement made by León-Carrión (1998): “*Different authors, apart from Santiago Ramón y Cajal, can be considered founders of Spanish neuroscience and neuropsychology, such as Cubí, Simarro, Lafora, Gonzalo, and Lorente de Nó*” (translated from Spanish).

It could be said that Gonzalo was decades ahead of subsequent discoveries. In his working notes, he even anticipated that it would take time for other authors to reach certain results and that, in general, his conclusions would be better understood in the 21st century. Indeed, this has proved to be the case with the later proposal of cortical gradients, with the highly active research that now considers such gradients as fundamental, and with the extensive studies on multisensory processes that have proliferated since around 2000, as noted in the Introduction. Yet much of his work, though of continuing relevance, remains largely unknown. As García-Molina (2024) noted in a review of the 2023 English edition of Gonzalo’s work, “*its content is innovative in many aspects, and its relevance persists today*.”

## Data Availability

The raw data supporting the conclusions of this article will be made available by the authors, without undue reservation.

## Footnotes

1 Letter from Henri Piéron (Collège de France) to Gonzalo, dated November 26, 1945, held in the Justo Gonzalo Archive, Cajal Institute, Madrid.

2 Letter from Robert Bing (University of Basel, Switzerland) to Gonzalo, dated Abril 19, 1946, held in the Justo Gonzalo Archive, Cajal Institute, Madrid.

3 Letters from Wolfgan Köhler (Swarthmore College, United States) to Gonzalo, dated May 21, 1946 and on July 12, 1951, held in the Justo Gonzalo Archive, Cajal Institute, Madrid.

4 Letters from G. Rodríguez Lafora to Gonzalo, written in Mexico on April 8 and 20, 1946, held in the Justo Gonzalo Archive, Cajal Institute, Madrid.

5 Letter from Carlos Jiménez Díaz (Madrid) to Gonzalo, dated December 27, 1950, held in the Justo Gonzalo Archive, Cajal Institute, Madrid.

6 Letter from Eduardo Tolosa Colomer (Barcelona) to Gonzalo, dated Mars 5, 1950, held in the Justo Gonzalo Archive, Cajal Institute, Madrid.

7 Letter from Gregorio Marañón (Madrid) to Gonzalo, dated November 6, 1954, held in the Justo Gonzalo Archive, Cajal Institute, Madrid.

8 Letter from Carlos Castilla del Pino (Córdoba) to Gonzalo, dated December 1, 1954, held in the Justo Gonzalo Archive, Cajal Institute, Madrid.

9 *Apuntes de Fisiopatología Cerebral. Extractos de las lecciones del profesor Dr. J. Gonzalo*, (Notes on Brain Pathophysiology: Excerpts from the Lectures of Professor Dr. J. Gonzalo) por los doctores C. Paumard y R. Thomas. Cursos 1952–53 y 1955–56, Facultad de Medicina, Madrid. 104 pages. Other noteworthy class notes are from P. Sánchez Mesón (course 1957–58), and from O. Delgado Schwatz (course 1965–66). Held in the Justo Gonzalo Archive, Cajal Institute, Madrid.

10 See letters from L. M. Hernando de Larramendi (New York) to Gonzalo, dated Mars, 21 and April, 08 in 1957, held in the Justo Gonzalo Archive, Cajal Institute, Madrid.

11 Comments from former students of the doctoral course on Brain Pathophysiology. Faculty of Medicine, Madrid. 1954–1966. Held in the Justo Gonzalo Archive, Cajal Institute, Madrid.

12 Document from the Dean of the Faculty of Medicine of Madrid to Justo Gonzalo on October 11, 1966, held in the Justo Gonzalo archive, Cajal Institute, Madrid.

13 Letter to the Dean of the Faculty of Medicine in Madrid signed by V. Gilsanz, R. Vara López, C. Jiménez Díaz, F. Orts Llorca, and J. Botella Llusiá (vice Dean). Dated November 28, 1966. Held in the Justo Gonzalo Archive, Cajal Institute, Madrid.

14 Letter from Erol Başar (Medizinische Universität zu Lübeck) to the daughters of Justo Gonzalo, dated July, 16, 1991, held in the Justo Gonzalo Archive. Cajal Institute, Madrid.
